# Perceptions and Practices of Saudi Hospital Pharmacists Towards Reporting Medication Errors Including Near Misses

**DOI:** 10.7759/cureus.51987

**Published:** 2024-01-10

**Authors:** Abdullah Al Hamid

**Affiliations:** 1 Pharmacy Practice, King Faisal University, Al Ahsa, SAU

**Keywords:** attitude of patients, patient safety, pharmacists, near misses, medication errors

## Abstract

Objectives: Medication errors (MEs) represent a patient safety concern that can have negative consequences on patients in the short and long term. Community pharmacists play an important role in the medication management process, which urges the need for their role in managing MEs. Therefore, this study aimed to investigate the perceptions and attitudes of Saudi pharmacists towards reporting MEs.

Methodology: A cross-sectional study was conducted using a semi-structured questionnaire that was distributed to Saudi pharmacists. The questionnaire was distributed to pharmacists via email after they had provided their consent to take part in the study. Data from the questionnaire were analysed using Statistical Product and Service Solutions (SPSS) (IBM SPSS Statistics for Windows, Armonk, NY), where descriptive statistics were applied.

Results: The findings showed that most pharmacists appreciated the importance of reporting MEs and the role the reporting played in improving the quality of healthcare delivery. However, pharmacists raised many concerns regarding barriers to reporting. Such barriers to reporting included blaming patients or healthcare professionals, underdeveloped protocols, and the lack of standard procedures for ME reporting. Moreover, inadequate communication between healthcare professionals (for example, between pharmacists and doctors) represented an additional barrier to reporting MEs.

Conclusions: MEs and near misses are underreported among Saudi pharmacists due to many operational and communication challenges. These findings are useful for healthcare authorities involved in developing patient safety frameworks for reporting MEs and near misses. Future work can also determine the attitudes of other healthcare professionals involved in the medication management process.

## Introduction

Patient safety is defined as the freedom of harm to patients [1]. Medical negligence from healthcare professionals affects patient safety. An example of medical malpractice is medication errors (MEs), which are defined as errors encountered during the prescription, dispensing, or intake of medicines [2]. The United States National Coordinating Council for Medication Error Reporting and Prevention (NCC MERP) defines MEs as preventable incidents in which patients face problems because of the wrong prescription or administration of medicines by healthcare professionals [3].

MEs result in increased hospitalisation and increased economic burden on the healthcare system [4]. The most common MEs reported include incorrect or incomplete regimens, incorrect dosing, the presence of DDIs, and incorrect administration [5].

MEs are classified into four broad categories related to knowledge-based errors, rule-based errors, action-based errors, and memory-based errors [6]. Knowledge-based errors are related to healthcare professionals’ knowledge of medicines [7,8]. Action-based and memory-based errors are more related to decision-making [6-8]. Hence, preventing MEs requires the knowledge and experience of pharmacists in medicines that are necessary for patients’ optimal outcomes [8].

In this respect, education of pharmacists is essential in preventing MEs, and many initiatives have been developed in this sense [9-12]. Education creates continuous learning and open communication that allows reporting errors in a blame-free environment [11,12]. In this respect, not only knowledge and actions are important for preventing MEs but also reporting plays a key role in early prevention [9]. Hence, encouraging the reporting of MEs is essential for reducing MEs and creating a safety culture [12]. Likewise, reporting near misses offers early prevention of failures that are potential causes of MEs [13]. Near misses are defined as errors that have the potential to cause an adverse event to patients but do not cause it due to an accident [13].

However, many barriers exist relating to reporting MEs and have been highlighted in several studies [14-18]. These included a lack of awareness of healthcare professionals regarding reporting systems [14,15], insufficient knowledge about the concept of MEs [15,16], fear of punitive actions as a consequence of reporting [17,18], and a lack of proper procedures for reporting [19].

Within Saudi Arabia, there is an increased focus on implementing a patient safety culture; however, some aspects relating to patient safety need development, such as MEs reporting [20,21]. MEs in Saudi are often underreported and where reported they ranged between 40% and 70% [11]. Thus, there is still no clear mechanism for reporting MEs, especially in hospitals. Prior to reporting errors, it is essential to measure healthcare professionals’ knowledge and attitudes towards MEs and patient safety. Therefore, the present study determined this aspect by investigating Saudi hospital pharmacists’ knowledge of MEs and near misses and their attitudes towards reporting MEs and near misses. Therefore, the present study determined this aspect by investigating Saudi hospital pharmacists’ perceptions and attitudes towards reporting MEs and near misses. In this sense, this was measured by distributing a questionnaire survey to pharmacists in public and private hospitals.

## Materials and methods

Study settings and participants

The study comprised a semi-structured questionnaire survey of both open- and close-ended questions that was disseminated to included pharmacists via email. The questionnaire was disseminated between February and May 2023. Included pharmacists were those working at Saudi hospitals in the Southern region of Saudi Arabia and who were registered at the Saudi Ministry of Health were involved in the study. All areas (specialities) relating to hospital pharmacy practice were targeted [2,14]. Hospitals were targeted in this study considering the serious consequences that patients could face in hospitals.

Prior to disseminating the questionnaires, pharmacists were invited via email to participate in the study and were sent information about the study. Upon consent to take part, pharmacists were sent the informed consent form and the questionnaire via email. Pharmacists completed both forms offline and sent them back via email as attachments. They were followed up with an email reminder two weeks after the initial email.

Questionnaire development and distribution

The questionnaire was developed based on Kang et al.’s (2017) questionnaire that investigated Korean hospital pharmacists’ perceptions of MEs and near misses [22]. The literature relating to MEs and near misses was also considered during the questionnaire development. In this respect, literature studies relating to pharmacists' knowledge, facilitators, and barriers to reported errors were envisaged to support the questionnaire development [14].

The questionnaire explored six areas relating to (1) demography; (2) pharmacists’ knowledge, perceptions, and attitudes of reporting MEs and near misses; (3) pharmacists’ experiences and incidences of MEs and near misses; (4) MEs and near misses reporting rates; (5) pharmacy system for reporting MEs and near misses; and (6) training on MEs and near misses.

The content validity of the questionnaire was assessed by five Saudi hospital pharmacists who had extensive experience in ME reporting [18]. The content validity index was done for all items where the experts scored content validity indices in the range of between 0.8 and 1. The scale content validity index obtained was 0.95 and that confirmed the questionnaire’s content validity. It further confirmed that its items were clear and understandable. Moreover, the questionnaire’s validity and reliability were tested by 20 Saudi hospital pharmacists who were not included in the main study. Cronbach’s alpha value obtained in this case was above 0.8 for all questionnaire items and that indicated a good internal consistency.

List of definitions

MEs are defined as preventable errors resulting from inappropriate use of medicines that can result in harm to patient(s). MEs can result at any stage of the medicine lifecycle [23]. The ME severity rate is classified into seven levels, of which level 0 represents no error and level 6 has lethal effects. Near misses are provisional errors that do not cause an ME and that are detected before the error occurs and/or before medicine reaches the patient(s) [24].

Data analysis

Prior to data analysis, data obtained from the questionnaire were checked for completeness in Microsoft Excel version 2016. Then, the completed data were transferred to Statistical Product and Service Solutions (SPSS, v22) (IBM SPSS Statistics for Windows, Armonk, NY). Data analysis was conducted using SPSS, where descriptive statistics were applied. Descriptive statistics involves calculating frequencies and percentages for categorical variables and means/medians for continuous variables. Results were reported for each variable considering the categories obtained from respondents. In this respect, frequencies and percentages were reported under each category.

## Results

Participant characteristics

A total of 91 pharmacists out of 300 completed the questionnaire. Of these 91 pharmacists, 72 (82.4%) were males, and 16 (17.6%) were females (Table 1). The age of the participants was mainly below 40 years old. Hence, 40 (43.9%) participants were in the age range of 31-40 years, followed by 37 (40.7%) participants in the age range of 21-30 years. In contrast, only one participant was in the age range above 60 years old. The educational background of the participants showed that 48 (53.3%) had a bachelor’s degree, representing more than half of the participants. On the other hand, fewer participants had high degrees (master's or PhD) (Table 1). Eighty-seven (95.6%) participants were employed full time. In addition, most participants (32.9%) had two to five years of experience, whereas 35 (32.9%), 25 (27.5%), and 20 (21.9%) had two to five years, 5-10 years, and 10-20 years of experience, respectively. However, only eight (8.79%) had less than one year or more than 20 years of experience. Most of the participants worked in the public sector and included 68 (75.6%) of the participants.

**Table 1:** Characteristics of pharmacists that participated in the study. NA: Not applicable

Characteristic	Category	N (%)
Gender	Male	72 (82.4%)
	Female	16 (17.6%)
	NR	3 (3.29%)
Total	NA	91
Age range	21-30	37 (40.7%)
	31-40	40 (43.9%)
	41-50	9 (9.89%)
	51-60	4 (4.4%)
	60+	1 (1.1%)
Total	NA	91
Education level	Diploma	18 (20%)
	Bachelor	48 (53.3%)
	Masters	12 (13.3%)
	PhD	7 (7.78%)
	Other	5 (5.56%)
	NR	1 (1.09%)
Total	NA	91
Full-time employment	Full time	87 (95.6%)
	Part time	4 (4.39%)
Total	NA	91
Experience (years)	< 1	8 (8.79%)
	2-5	35 (32.9%)
	5-10	25 (27.5%)
	10-20	20 (21.9%)
	> 20	3 (3.29%)
Total	NA	91
Sector	Public	68 (75.6%)
	Private	22 (24.4%)

Knowledge of participants of medication errors and near misses

When asked about knowledge of MEs, 76 (83.5%) participants expressed knowledge about MEs (Table 2). On the other hand, fewer participants knew about near misses, with 60 (65.9%). When asked about the sources of knowledge of MEs, more than one-third of participants reported hospital work as the source of knowledge about MEs and represented (33, 43.4%) of participants. This was followed by 24 (31.6%) participants who sourced their knowledge from college. Furthermore, 13 (31.5%) participants indicated that they shared their knowledge from college, and six (7.89%) stated that they gained it at an academic conference.

**Table 2:** Knowledge of participants about medication errors and near misses. NA: not applicable NR: not reported

Parameter	Category	N (%)
Knowledge about ME	Yes	76 (83.5%)
	No	15 (16.5%)
Total	NA	91
Knowledge about near misses	Yes	60 (65.9%)
	No	16 (17.6%)
	NR	15 (16.5%)
Total	NA	91
Sources of knowledge about MEs	College	24 (31.6%)
	Hospital pharmacist training	13 (17.1%)
	During the course of hospital work	33 (43.4%)
	In an academic conference	6 (7.89%)
	NA	15 (16.5%)

MEs and near misses reporting

When asked about reporting MEs, approximately half of the participants claimed they had reported MEs, while the others did not report MEs or did not answer the questions (Table 3). In addition, 57 (62.6%) participants claimed that the importance of reporting MEs is extremely helpful to patients, whereas approximately 40% of participants found MEs reporting not so helpful. Although numerous participants stated that MEs were helpful, only 23 (25.3%) and 21 (23.1%) participants perceived that the level of reporting was extremely effective or very effective in preventing MEs, respectively. Furthermore, more than half of the participants expressed no barriers to reporting MEs. However, their response varied in relation to reporting depending on whether they were involved in the incident or not. In this respect, participants were more likely to report an incident if they were involved in it. Hence, 53 (58.2%) and 16 (17.6%) of the participants stated that they would report an incident they were involved in. However, 34 (37.4%) and 33 (43.4%) participants were very likely or likely to report incidents, respectively, if they were not involved.

**Table 3:** Reporting MEs and near misses among pharmacists. NA: Not applicable NR: Not reported

Parameter	Category	N (%)
Reporting MEs and near misses	Yes	52 (57.1%)
	No	24 (26.4%)
	NA	15 (16.5%)
Total	NA	91
Importance of reporting MEs and near-misses	Extremely helpful	57 (62.6%)
	Helpful	2 (2.19%)
	Somewhat helpful	16 (17.6%)
	Not so helpful	16 (17.6%)
Total	NA	91
Perception of the level of reporting activity in preventing MEs	Extremely effective	23 (25.3%)
	Very effective	21 (23.1%)
	Somewhat effective	27 (29.7%)
	Not so effective	5 (5.49%)
	Not at all effective	1 (1.09%)
	NR	37 (40.7%)
Total	NA	91
Barriers to reporting MEs and near-misses	Yes	26 (28.6%)
	No	51 (56%)
	NR	15 (16.5%)
Total	NA	91
Likelihood of reporting an incident not involved in	Very likely	34 (37.4%)
	Likely	33 (43.4%)
	Unlikely	7 (7.69%)
	Very unlikely	0
	NR	17 (18.7%)
Total	NA	91
Likelihood of reporting an incident involved in	Very likely	53 (58.2%)
	Likely	16 (17.6%)
	Unlikely	3 (3.29%)
	Very unlikely	1 (1.09%)
	NR	18 (19.8%)

Training on MEs and near misses

When asked about training in ME, 45 (49.5%) of the participants reported receiving training in MEs in the hospital (Table 4). For reporting MEs in hospital settings, electronic systems were often used for both MEs and near misses and were claimed by 43 (47.3%) participants. Difficulty in reporting MEs was described as easy and fast by 37 (40.7%) participants and complicated and lengthy by 34 (37.4%) participants. Almost half of the participants claimed that there was legal protection post-reporting. Nonetheless, only 19 (20.9%) of the participants stated that ME reporting had a negative impact on pharmacists.

**Table 4:** Pharmacists' training regarding MEs and near misses. NA: Not applicable NR: Not reported

Parameter	Category	N (%)
Training in MEs in the hospital	Yes	45 (49.5%)
	No	25 (27.5%)
	NA	21 (23.1%)
Total	NA	91
Using an electronic system for reporting MEs and near misses	Yes	43 (47.3%)
	No	27 (29.7%)
	NR	21 (23.1%)
Total	NA	91
Difficulty of ME reporting procedure	Easy and fast	37 (40.7%)
	Complicated and lengthy	34 (37.4%)
	NR	20 (21.9%)
Total	NA	91
Presence of legal protection post-reporting	Yes	45 (49.5%)
	No	24 (26.4%)
	NR	22 (24.2%)
Total	NA	91
Negative impact of reporting on pharmacists	Yes	19 (20.9%)
	No	50 (54.9%)
	NR	22 (24.2%)

## Discussion

Medical negligence is a public health problem that impacts patients globally [25]. MEs are a subset of medical negligence that has an impact on patients globally [4]. The causes and risk factors contributing to medical errors vary and could be related to the patient, healthcare professionals, healthcare systems, and/or the medicine(s) [26]. The co-occurrence of the aforementioned factors contributes to an increased incidence of MEs, such as a lack of knowledge/decreased knowledge among healthcare professionals or reduced communication between healthcare professionals [27]. In this respect, reduced communication could be attributed to different backgrounds and educational systems [28].

Saudi Arabia is a country with a diverse healthcare system that has healthcare professionals from different countries with different experiences and education levels [29]. In addition, many Saudi hospitals have shortages of pharmacy staff, which increases the workload on pharmacy staff [29]. Increased workload in such cases results in a lack of focus of pharmacists, which can result in MEs [20]. Moreover, differences in educational backgrounds contribute to a lack of or decreased communication between pharmacists and doctors [29]. This in turn contributes to dispensing the wrong medicines to patients.

Subsequently, the present study explored the knowledge and attitudes of hospital pharmacists in Saudi Arabia towards MEs and near misses. Pharmacists participating in this research were mainly (> 80%) with a bachelor’s degree or above, with more than two years of experience and mainly working in the public sector (75.6%). The majority of pharmacists (82.4%) were male, which was consistent with other studies in the literature that showed a majority of males [15]. This is understandable from the fact that most of the pharmacists working in the Kingdom are males. Moreover, females are mostly reluctant to take part in activities such as surveys and interviews because of cultural constraints in Saudi Arabia [11].

Pharmacists in the present expressed more knowledge about ME but not near misses. However, only 57.1% of pharmacists reported MEs, and 63% found reporting effectiveness. Additionally, 58.2% of pharmacists were more likely to report an incident in which they were involved. These findings were consistent with findings in other studies that stated that patients had not reported MEs due to concerns about taking blame from patients, colleagues, or hospitals [11]. This finding urges the need for hospital management to facilitate positive and non-blame cultures for reporting MEs.

Likewise, the reporting of near misses was low, which could be linked to the lack of knowledge and/or decreased knowledge of near misses [21]. Hence, most pharmacists in this study received education about MEs and near misses in hospitals or during the course of hospital work, which may not be deemed sufficient in the presence of a high workload [28,29].

Nonetheless, in both MEs and near-misses cases, pharmacists expressed their appreciation of the importance of reporting MEs and near misses [20]. However, many pharmacists were not aware of the reporting systems in place. Therefore, it is important for hospitals to educate pharmacists about the reporting system in their country, including mechanisms and procedures for reporting, as well as the ‘blame-free’ environment for reporting.

Encouraging reporting in a ‘blame-free’ environment contributes to a patient safety culture where patients receive the optimal therapy and desired health outcomes [14]. In this respect, other studies have assured the importance of reporting errors alongside communication and teamwork across hospitals, especially in multicultural environments [12]. Reporting MEs at the right time allows for taking immediate actions that save patient lives and are of economic benefit to the healthcare system [12]. This should be supported by organisational policies that can support reporting incidents, including MEs and near misses. Furthermore, organisational resources should be sufficient for reporting MEs on a regular basis. This in turn will provide pharmacists with facilities and systems for reporting near misses and MEs early, thereby enabling early prevention of MEs.

A few limitations were encountered in this study and should be highlighted. The first was related to the sample size, which was small compared to quantitative studies. The respondents in the study were hospital pharmacists working in Saudi Arabia. Therefore, the findings are not generalisable to community pharmacists or pharmacists working in different countries. Moreover, there could be unreported MEs and near misses because pharmacists would report only MEs they were aware of. Thus, there could have been a potential for ‘recall bias’. Moreover, some MEs/near misses could be underreported due to ‘fear of blame’. However, since the study reported MEs over a wide range of specialities within the hospital, underreporting would not have a large impact to affect the results.

## Conclusions

The findings of the present study highlighted the need for a reporting system in Saudi Arabia and the importance of reporting in improving healthcare services. Most pharmacists were aware of the importance of reporting; however, not all the time pharmacists reported errors due to fear of consequences. Moreover, pharmacists showed limited knowledge about near misses, which was related to increased working hours and a lack of sufficient training. This urges the need for hospitals and healthcare systems to provide a patient safety framework that includes procedures and mechanisms for reporting errors in a blame-free environment. A patient safety framework will in turn improve the patients’ quality of life, public health, and the country’s economy. Therefore, future work should build on the findings of the study in exploring approaches to implementing patient safety culture in hospitals in Saudi Arabia.
